# Organization of Post-Transplant Care and the 5-Year Outcomes of Kidney Transplantation

**DOI:** 10.3390/ijerph19042010

**Published:** 2022-02-11

**Authors:** Agnieszka Szymańska, Krzysztof Mucha, Maciej Kosieradzki, Sławomir Nazarewski, Leszek Pączek, Bartosz Foroncewicz

**Affiliations:** 1 Department of Immunology, Transplantology and Internal Diseases, Medical University of Warsaw, 02-091 Warsaw, Poland; agnieszka.szymanska1@wum.edu.pl (A.S.); krzysztof.mucha@wum.edu.pl (K.M.); leszek.paczek@wum.edu.pl (L.P.); 2 Institute of Biochemistry and Biophysics, Polish Academy of Sciences, 02-091 Warsaw, Poland; 3 Department of General and Transplantation Surgery, Medical University of Warsaw, 02-091 Warsaw, Poland; maciej.kosieradzki@wum.edu.pl; 4 Department of General, Vascular and Transplant Surgery, Medical University of Warsaw, 02-091 Warsaw, Poland; slawomir.nazarewski@wum.edu.pl

**Keywords:** comorbidities, follow-up, kidney transplantation, organization, outcome

## Abstract

The outcomes of kidney transplantation depend on numerous factors and vary between transplant centers. The aim of this study is to assess the relationship between selected organizational factors, comorbidities, and patient and graft survival. This is a retrospective analysis of 438 renal transplant recipients (RTR) followed for 5 years. Patient and graft survival were evaluated in relation to hospitalization length, distance from the patient’s residence to the transplant center, the frequency of outpatient transplant visits, and the number and type of comorbidities. Five-year patient and graft survival rates were 93% and 90%, respectively. We found significant associations of patient survival with the prevalence of pre-transplant diabetes, cardiovascular diseases, malignancies, the number of comorbidities, and the first post-transplant hospitalization length. The incidence of infections, cardiovascular diseases, and transplanted kidney diseases was 60%, 40%, and 33%, respectively. As many as 41% of RTR had unknown etiology of primary kidney disease. In conclusion, the organization of post-transplant care needs to be adapted to the multi-morbidity of contemporary RTR and include multi-specialist care, especially in the context of current problems related to the COVID-19pandemic. The high proportion of patients with undetermined etiology of their primary renal disease carry the risk for additional complications during their long-term follow-up.

## 1. Introduction

Kidney transplantation (KTx) is the best available method of treatment for end-stage kidney disease (ESKD). Its outcomes depend on numerous factors and vary between con tinents, countries, and sometimes between transplant centers [1,2,3]. Renal transplant recipients (RTR) are a particularly sensitive group of patients, due to the multitude of risks and complications associated with comorbidities and immunosuppression (IS). Of course, the IS protects against the acute and chronic graft rejection [4,5], but, on the other hand, it also increases the risk of cardiovascular diseases (CVD), malignancies, and infections, which are the leading causes of RTR deaths [6,7,8]. As we know, contemporary people live longer, mostly because previously lethal diseases became curable thanks to the continuous development of medical care. As a result, the number of RTR is also increasing and requires tailored, holistic long-term post-transplant care [9,10,11]. It must be added that there are many so-called “soft” (often immeasurable, environmental, and/or psycho-social) factors that might affect the long-term outcomes of KTx. One of them is nonsteroidal anti-inflammatory drugs (NSAIDs) and over-the-counter (OTC) analgesics abuse. It is well known that these drugs are commonly used and may cause numerous adverse effects, including gastrointestinal diseases, nephro-toxicity, and hepato-toxicity. RTR are at high risk of these toxicities. We reported previously that 63% of RTR declared regular use of OTC painkillers and 30% of RTR were unaware of the potential side effects [12]. We also found that 44% of RTR took dietary supplements and/or herbal products, usually without medical consultation [13]. Importantly, both groups of products can interfere with IS [14]. Other psycho-social variables that may influence follow-up include alcohol abuse and non-compliance [15,16]. The alcohol consumption among RTR occurred in 0.014 cases per year, but its correlation with patient or graft survival remains unknown [15]. Appointment and IS non-adherence were highly correlated, and both were significant independent predictors of the worse outcomes [17]. Additionally, environmental factors, including stress and exposure to metals (such as cadmium, lead, and arsenic) or parasites, may be associated with renal function [18,19,20]. In the recent COVID-19 pandemic era, SARS-Cov-2 infection resulted in different outcomes in RTR than in the general population, and also required different treatment and prevention strategies [21]. The results of the available studies necessitate consideration of these associations with respect to prevention, diagnostics, monitoring, and management.

For these reasons, the optimization of care practices is essential for delivering adequate care to RTR. The importance of multi-disciplinary integration of patient care is generally accepted in the transplant society [22]. However, despite multiple regulations [23], the variation between transplant centers in approaches to donor and recipient evaluations, in-patient health care delivery, treatment team composition, coordination of care, relationship and communication between medicine and surgery teams, and frequency of follow-up were reported [24]. For example, the results of a survey of 156 transplant centers in the United States demonstrated that 65% of them do not have a dedicated transplant pharmacist in outpatient care, two-thirds do not see RTR at least monthly during the first year, and only less than 30% has an established nephrology and transplant surgery collaboration [24].

The aim of this study is to assess the relationship between selected organizational factors, comorbidities, and 5-year patient and graft survival in a single center.

## 2. Materials and Methods

This was a retrospective analysis of 438 consecutive adult kidney recipients transplanted between 2005 and 2012 in two surgical departments (SC1 and SC2). Their post-transplant care was centralized in the Department of Immunology, Transplantology, and Internal Medicine [25]. The duration of follow-up was 5 years. The patient and graft survival were evaluated in relation to the surgical center where the KTx was performed, the length of the first hospitalization after transplantation, the number of days of hospitalizations per patient per year, the distance of the patient’s residence from the transplant center, and the frequency of outpatient transplant visits. The number and type of comorbidities which could entail organizational adjustments were also assessed and grouped into 6 categories: (1) infections; (2) cardiovascular diseases; (3) transplanted kidney diseases; (4) new-onset diabetes after transplantation (NODAT); (5) diabetes mellitus (DM) before KTx; and (6) malignancies. Native kidney disease was an additional analyzed subject. Both the comorbidities and the primary renal diseases were related to the patient and graft survival and to the mentioned organizational factors. In addition, RTR transplanted in the years 2005–2008 and 2009–2012 were compared to check if the financial change in IS reimbursement influenced the outcomes. Namely, in 2008, there was a change in the national regulations of IS disposal. Before this date, patients received their drugs free of charge during the ambulatory visits from the dedicated pharmacy located at the transplant centers. After 2008, they started to buy IS in pharmacies, independent from their out-patient visits. The drug costs remained covered by the government, but the coverage dropped from 100 to 90–95% depending on the drug types.

The study source of the data was in-patient and out-patient medical records. The study was approved by the Ethical Committee (#AkBF/103/2015 from the 8 September 2015). In this study design, no patient informed consent was required.

### Statistical Analysis

The continuous variables in the analysis were normally distributed, as verified using the Shapiro–Wilk test. A linear regression (r) was used to investigate the correlation between variables. The differences between means and frequency of results in individual groups were checked using Mann–Whitney (t) and chi-square (chi) tests. The Kaplan–Meier curve was used to assess the 5-year patient and graft survival. In order to compare the survival curves and indicate possible differences between the groups, the standard Fleming–Harrington test (weighted log-rank test) was used. The *p*-value of less than 0.05 was considered significant. The statistical analysis and visualization were conducted using R Core Team (2020) (R: A language and environment for statistical computing. R Foundation for Statistical Computing, Vienna, Austria).

## 3. Results

### 3.1. Patients and Their Survival Rates

All 438 RTR were Caucasians in the mean (SD) age of 47.2 (13.3) years at the time of transplantation. The male to female ratio was 58:42. The mean (SD) donor age was 46.4 (13.8) years. The most frequent indications for kidney transplantation included: (1) autosomal dominant polycystic kidney disease (ADPKD); (2) glomerulonephritis (GN); (3) tubulointerstitial nephritis (TIN); and (4) diabetic nephropathy (DN). The diagnosis was not determined prior to transplantation in as many as 41% of patients (Table 1).

**Figure 1:**
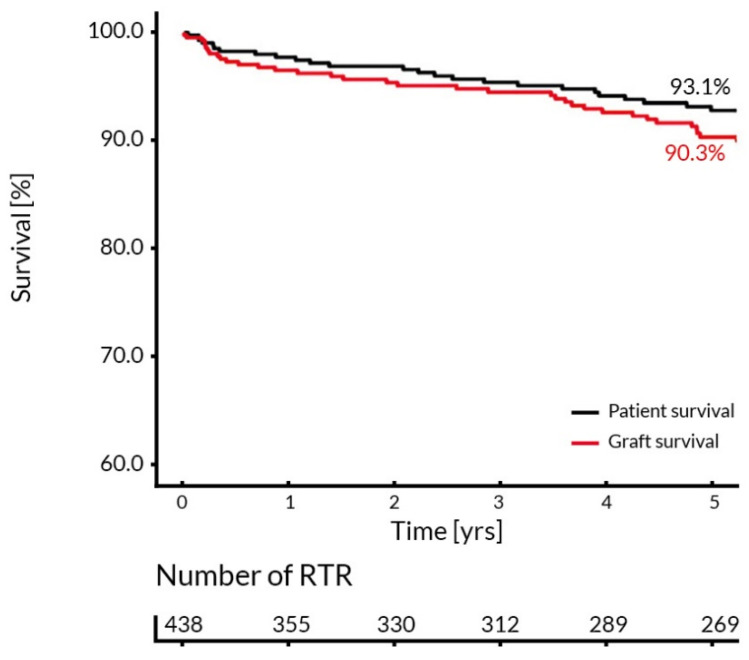
Kaplan–Meier estimates of the 5-year patient and graft survival in the entire studied transplant population.

In total, 80% of the studied population were recipients of the first transplanted kidney, 16.6% had a second transplantation, and the remaining 3.4% of RTR underwent third or fourth kidney transplantations. An amount of 52% of the patients completed the entire 5-year follow-up. Among the remaining 48% of RTR: 7% died; 12.3% lost their graft and returned to dialysis; 21.7% relocated to another transplant center; and in 7.3% the reason for a lack of follow-up could not be determined. The patient and graft survival curves are presented in Figure 1.

### 3.2. Organization of the Post-Transplant Care

**Figure 2:**
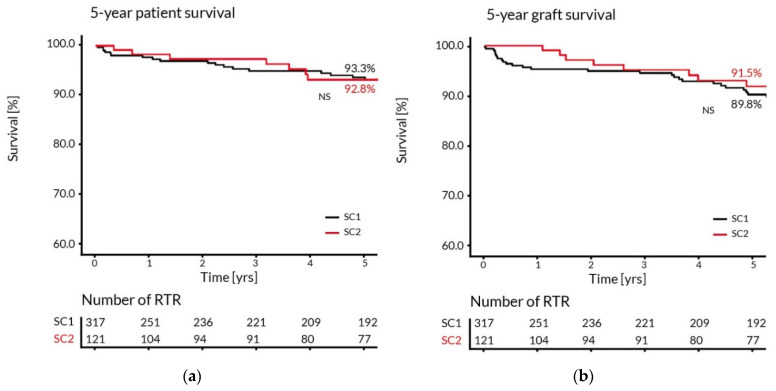
The Kaplan–Meier estimates of the 5-year patient (**a**) and graft (**b**) survival in RTR transplanted in two surgical centers (SC1 and SC2).

The 5-year follow-up of RTR transplanted in two different surgical centers revealed no significant differences in patient and graft survival (Figure 2).

**Figure 3:**
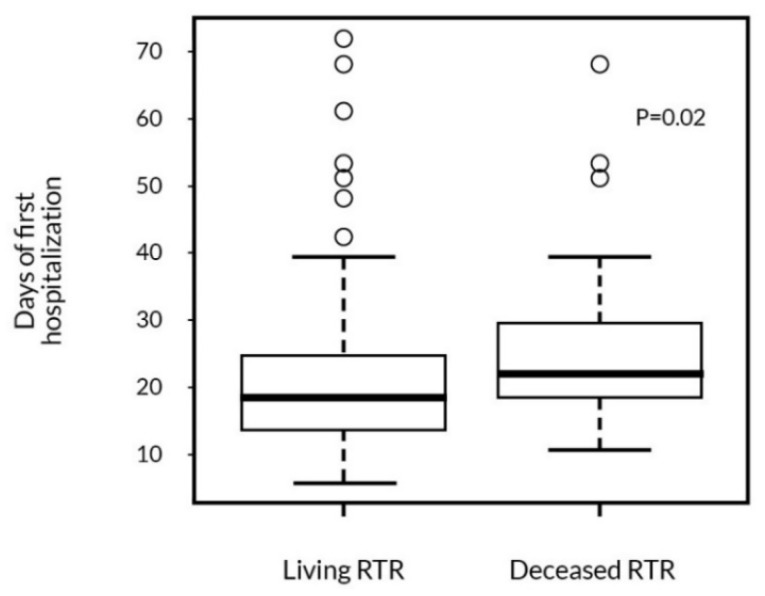
The association of patient survival with the length of the first hospitalization (Mann-Whitney test).

The average length of the peri-transplant hospitalization was 23 (±16) days. It was not statistically different between the two surgical centers: 22 ± 18 vs. 26 ± 11 days. During later post-transplant follow-up, patients were hospitalized 10 days per year on average. Interestingly, 28% of RTR were only hospitalized during their first hospitalization after the KTx. We found a significant association of the first hospitalization length with patient survival. RTR who died during follow-up had a significantly longer peri-transplant hospitalization time (Figure 3).

**Figure 4:**
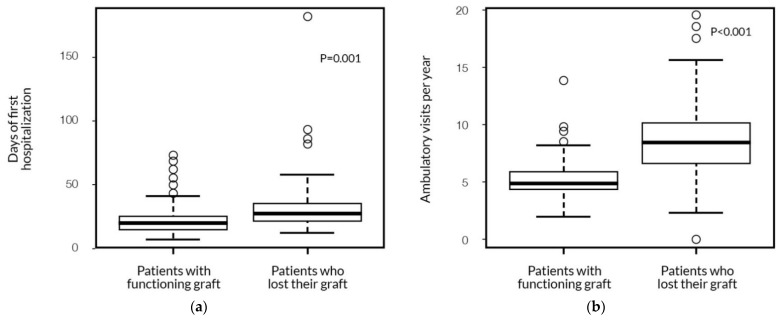
The association of graft survival with the first hospitalization length (**a**) and with the number of outpatient visits (**b**) (Mann–Whitney test).

The patients who lost their graft during follow-up and returned to dialysis also had a significantly longer first hospitalization length after transplant surgery (33 ± 27.4 days), compared to the patients with a functioning graft (20 ± 10.5 days). They required more outpatient visits than the patients with the functioning grafts: 9 ± 4.2 vs. 5 ± 1.4 per year, respectively (Figure 4).

**Figure 5:**
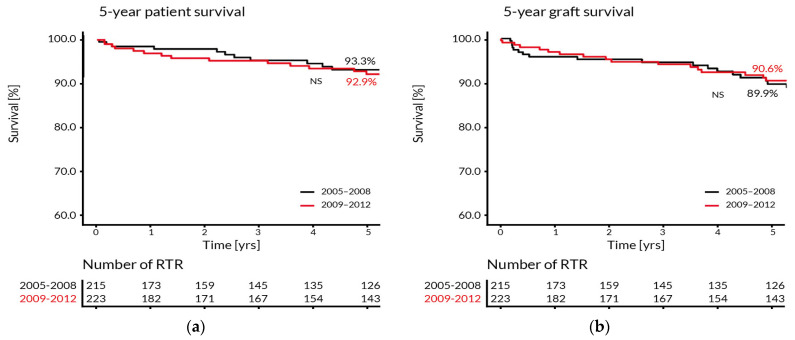
The Kaplan–Meier estimates of the 5-year patient (**a**) and graft (**b**) survival in two transplantation periods: 2005–2008 and 2009–2012.

Interestingly, patients with ADPKD required significantly less hospitalization days than RTR with other primary diseases (5.2 vs. 11.8 days per year, *p* = 0.003). These patients were hospitalized the shortest (5 days per year, on average) and had the least number of outpatient visits (6 visits per year, on average). The average distance from the patient’s place of residence to the transplant center was 186.5 km. The most remote location was 521 km away from the center and only 12.5% of the patients lived in the city where the transplant center is located. However, the distance from the place of residence to the transplant center was neither associated with the patient and graft survival, nor affected the number of outpatient transplant visits per year. The regulation of drug disposal in 2008 determined the reduction of the number of patient visits from 9.6 to 6.6 per year in the periods 2005–2008 and 2009–2012, respectively. Therefore, we compared the 5-year patient and graft survival in both periods and found no significant differences (Figure 5).

### 3.3. Comorbidities

The most frequent comorbidities diagnosed in renal transplant recipients were grouped into six major categories and are presented in Table 2.

Three of these categories significantly affected patient survival: CVD, malignancies, and pre-transplant DM. The patient survival rates in RTR diagnosed with these diseases were 77%, 66%, and 60%, respectively, and were significantly lower than in recipients without these comorbidities (Table 3).

**Figure 6:**
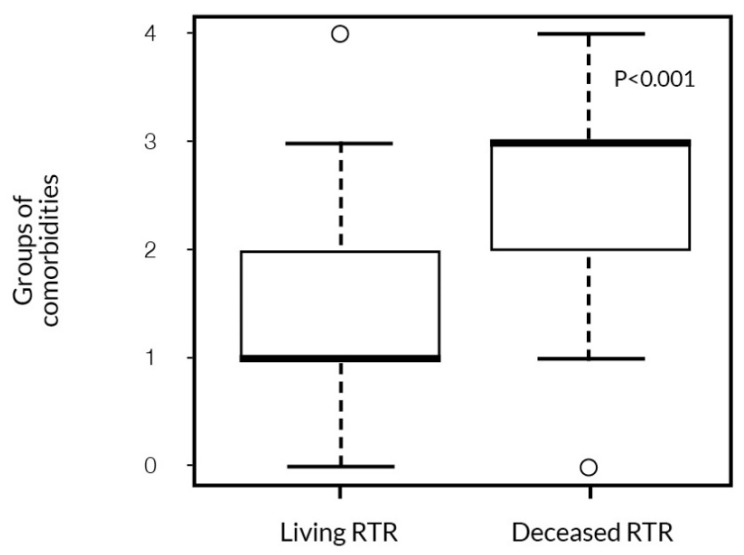
The association between the number of comorbidities and patient survival (Mann–Whitney test).

Importantly, we found that male gender had a significant impact on survival with these comorbidities. Diabetic nephropathy was the only primary disease found to be significantly associated with patient survival. Both the type and the number of categories of comorbidities influenced patient survival. RTR who had more comorbidities from different categories had significantly worse survival (Figure 6).

## 4. Discussion

The major finding of our study is the significant association of 5-year patient survival with the prevalence of pre-transplant diabetes, and the type and number of comorbidities. We did not find the influence of the allocation to a specific surgical center and the outcomes. These observations underline the importance of the quality of a multi-specialist system in long-term post-transplant care, enabling effective multi-morbidity management in RTR. These findings are also supportive to the shift from the short-term to the long-term post-transplant care paradigm observed in recent decades [26].

From the beginning of kidney transplant programs, RTR care has evolved based on a model in which clinical management focused around the surgical procedure and reflected the concept that perioperative and short-term interventions are primary determinants of success. New data emerging from major longitudinal graft outcome studies and registries highlighted the primacy of later events and comorbidities in influencing long-term outcomes [27,28,29,30]. Therefore, in the current era it is proposed that proper long-term care, a precise RTR monitoring system, and prompt diagnosis and treatment offer the best prospect of improving survival [26]. However, many transplant centers are held accountable by government and payers for performance metrics based largely on 1-year graft and patient survival [31]. Compared to the Western countries, many of whom incorporate different approaches to post-transplant care organization and reimbursement, outcomes in the United States are similar at 1 and 3 years, but demonstrate a widening decrement at 5 and 10 years [1,32]. For example, the 5-year survival of patients transplanted in the same time frame in Australia and New Zealand was higher than in Europe or the USA (90%, 87.1%, and 86.1%, respectively) [1,33,34,35]. The differences may be partially explained by the fact that in the US patients lost coverage for IS 3 years after transplantation, leading to non-adherence in some cases and late allograft loss [36]. More importantly, according to UpToDate recommendations, beyond 6–12 months post-transplantation patients may be followed-up by an internist or general nephrologist [37], however it was proven that very close life-long follow-up and adequate transplant care delivery to RTR improve their outcomes [38].

Numerous psycho-social and environmental factors may influence the outcomes of transplantation. Some of them are related not only to health, but also to socioeconomic status [39]. They were not analyzed in the current study, but they should be considered in the structure of the post-transplant care. Interestingly, we found that the distance from the patient’s place of residence to the transplant center neither influenced the number of ambulatory visits nor impacted the outcomes. Other authors also reported that RTR survival was not affected by the distance they had to travel to the transplanting center [40]. This finding suggests that it is rather the “on-site” organization of the post-transplant care than the location of the transplant center that contributes to the adequate care delivery. This should also be taken into consideration when organizing post-transplant care. In our transplant center, internal medicine physicians have additional specializations, including nephrology, transplantology, cardiology, diabetology, or gerontology. Their patients see them regularly every 3–4 months or more frequently if needed. A dietician and psychologist are also at patients’ disposal. We found that the reduction of the number of visits from 9.6 to 6.6 per year had no significant effect on their survival. Therefore, we believe that the +/− 6 transplant visits per year enables safe maintaining of the post-transplant care. Unfortunately, the study designed to verify this number is unfeasible in a single center. It would also be very difficult to quantitate the influence of physicians’ expertise on the outcomes. Nevertheless, the combination of such experience with the frequency of ambulatory visits, access to full-profile hospital diagnostics, and the established referral system to oncologists or surgeons might have contributed to the observed 93.1% patient survival rate in our study. The importance of establishing a referral system to improve the frequency and quality of post-transplant counselling was recently confirmed in the context of physical activity in Canadian RTR [41].

We are sure that a multi-disciplinary care organization is crucial for RTR, especially to those who suffer from comorbidities. Thanks to the multi-disciplinary care organization, most of the comorbidities identified in our study could be managed. Among them, we found pre-transplant DM to influence the outcomes the most adversely. This observation is in line with the results of previous studies. It was reported that RTR with pre-existing DM, coronary diseases, and peripheral vascular diseases showed a significantly inferior patient survival [6]. Moreover, their association with an increased risk of mortality was used in a predictive score for post-transplantation outcomes [42]. RTR with a past medical history of diabetes were more likely to be readmitted to a hospital within 30 days after the first post-transplant discharge and had lower adherence to their medical regimen [43]. The prevalence of pre-transplant DM in our study was much lower (8.4%) than reported in the US and this observation may partially explain better survival rates. Accordingly, previous studies have shown higher prevalence of recipient ESKD from diabetic nephropathy in the US than in other countries [1]. Ojo and colleagues reported 24.1% of ESKD from DN among RTR transplanted in the US vs. 5.6% in Spain [2]. Respectively, Gondos and others found it to be 26% in the US vs. 8.3% in Europe [3].

We observed that the first post-transplant hospitalization was longer in RTR who had more comorbidities, hence its length was associated with the 5-year survival. We did not analyze the impact of specific comorbidities on the hospitalization length, but it is well known that infections are the predominant cause of hospitalizations in the early post-transplant period [44,45]. Additionally, the CVD burden in RTR is both significant and increasing. It was reported that 30% of all post-transplant hospitalizations were linked to CVD [46].

Of note, we identified a need for improvement in the nephrological diagnostics before transplantation. The knowledge of primary kidney disease is of particular importance for patients with diseases that may reoccur and they could benefit from a different (i.e., more aggressive) approach. We found that the etiology of primary kidney disease remains unknown in as many as 41% of RTR. This percentage was lower than that observed in the years 1998–2003 in our center (58.4%) [47]. Despite the tendency to improve, there is a big space for improvement in the pre-transplant diagnostics.

Our study has several limitations. First, the study design was retrospective. Therefore, the detailed data on the reasons for prolonged first post-transplant hospitalization are lacking. The influence of the so-called “soft” factors, such as adherence to therapy, and alcohol or OTC drug use, that might interfere with the post-transplant therapy could not be measured with our study design. In fact, these psycho-social factors are hard to detect and measure in daily practice, and then correlate with patient and graft survival. It might be possible only using prospective study designs. Furthermore, due to regulatory limitations, we were unable to analyze the causes of death and follow-up of these patients who changed their transplant center. Finally, our cohort was homogenous in terms of race, and most recipients underwent cadaveric transplantation. As such, it may be difficult to compare some of our results with those obtained in different and more heterogeneous populations, for example from the United States, where most transplantations are from living donors.

## 5. Conclusions

In conclusion, the results of our study underline the need to adapt the organization of post-transplant care in transplantation centers to the multi-morbidity of contemporary RTR. Access to the multi-disciplinary transplant teams and the referral system to other specialists may improve patient safety and survival. RTR could also benefit from the improvement of pre-transplant nephrological diagnostics aimed at the identification of primary kidney disease etiology.

## Figures and Tables

**Table 1:** Causes of end-stage kidney disease in 438 renal transplant recipients.

Original Diagnosis	% of RTR	Number of RTR
Undetermined etiology	41	179
ADPKD	13	56
GN	10	44
TIN	9	39
DN	8	37
Other	19	83

Legend: ADPKD—autosomal dominant polycystic kidney disease; DN—diabetic nephropathy GN—glomerulonephritis; RTR—renal transplant recipients; TIN—tubulointerstitial nephritis.

**Table 2:** The most frequent comorbidities diagnosed in renal transplant recipients.

Infections	Cardiovascular Diseases	Transplanted Kidney Diseases	New-Onset Diabetes after Transplantation	Pre-Transplant Diabetes Mellitus	Malignancies
Cytomegalovirus	Hypertension	Transplant renal artery stenosis		Type 1 Diabetes	Multiple myeloma
Hepatitis C virus (HCV)	Atherosclerosis	Acute T-cell rejection		Type 2 Diabetes	Prostate cancer
Hepatitis B virus (HBV)	Coronary heart disease	Acute vascular rejection			Bladder cancer
Herpes Simplex viruses (HSV)	Myocardial infarction	Chronic antibody-mediated rejection			Acute myeloid leukemia
BK virus (BKV)	Ischemic stroke	Acute antibody-mediated rejection			Skin cancer
Urinary tract infections	Paroxysmal atrial fibrillation	Fluid collection near graft (lymphocele)			Colon cancer
Clostridium difficile colitis	Permanent atrial fibrillation	Vesicoureteral reflux			Lung cancer
Other infection colitis	Valve diseases	Acute renal failure			Pancreatic tumors
Bronchitis	Vein thrombosis	Drug nephrotoxicity			Hemangiomas
Pneumonia	Heart failure	Cystic degeneration of transplanted kidney			
Pulmonary tuberculosis	Cardiomyopathy				
Chronic sinusitis	Left ventricular hypertrophy				
Helicobacter pylori gastritis					
60.5%	39.5%	33%	14.4%	8.4%	7%

**Table 3:** The association of patient survival with the categories of comorbidities (Mann–Whitney test).

Groups of Comorbidities	All Patients			Females			Males		
	Survival of Patients with Disease [%]	Survival of the Remaining Patients [%]	*p*-Value (t)	Survival of Patients with Disease [%]	Survival of the Remaining Patients [%]	*p*-Value (t)	Survival of Patients with Disease [%]	Survival of the Remaining Patients [%]	*p*-Value (t)
Infections	87.20	90.32	0.363	89.61	94.44	0.357	85.06	87.72	0.649
**Cardiovascular diseases**	77.32	95.00	**0.000**	83.87	93.90	0.173	74.24	96.15	**0.000**
Transplanted kidney diseases	85.33	89.56	0.456	87.50	92.59	0.446	83.72	87.13	0.608
New-onset diabetes after transplantation	82.35	89.24	0.456	94.44	90.53	0.541	68.75	88.28	0.131
**Pre-transplant diabetes mellitus**	60.00	90.72	**0.000**	66.67	92.52	0.276	57.14	89.23	**0.038**
**Malignancies**	68.00	90.50	**0.003**	88.89	91.35	0.835	56.25	89.84	**0.020**

Statistically significant changes are bolded.

## Data Availability

Not applicable.
